# Passively Q-switched Ytterbium-doped fiber laser based on broadband multilayer Platinum Ditelluride (PtTe_2_) saturable absorber

**DOI:** 10.1038/s41598-019-46658-y

**Published:** 2019-07-12

**Authors:** Ping Kwong Cheng, Chun Yin Tang, Xin Yu Wang, Sainan Ma, Hui Long, Yuen Hong Tsang

**Affiliations:** 1 The Hong Kong Polytechnic University Shenzhen Research Institute, Shenzhen Guangdong, China; 2 Department of Applied Physics and Materials Research Center, The Hong Kong Polytechnic University, Hung Hom, Kowloon, Hong Kong, China

**Keywords:** Nonlinear optics, Fibre lasers

## Abstract

Two-dimensional (2D) layered Platinum Ditelluride (PtTe_2_), a novel candidate of group 10 transition-metal dichalcogenides (TMDs), which provides enormous potential for pulsed laser applications due to its highly stable and strong nonlinear optical absorption (NOA) properties. PtTe_2_ saturable absorber (SA) is successfully fabricated with firstly demonstrated the passively Q-switched laser operation within a Yb-doped fiber laser cavity at 1066 nm. Few layered PtTe_2_ is produced by uncomplicated and cost-efficient ultrasonic liquid exfoliation and follow by incorporating into polyvinyl alcohol (PVA) polymer to form a PtTe_2_-PVA composite thin film saturable absorber. The highest achieved single pulse energy is 74.0 nJ corresponding to pulse duration, repetition rate and average output power of 5.2 μs, 33.5 kHz and 2.48 mW, respectively. This work has further exploited the immeasurable utilization potential of the air stable and broadband group 10 TMDs for ultrafast photonic applications.

## Introduction

Q-switching, a useful and very important technique, has been widely studied and applied in pulsed laser development over the past decades^1–4^. Contributed by the remarkable high pulse peak power, Q-switched laser can be employed in vast applications, for instance, industrial laser engraving, nonlinear optics, skin treatment, and eyes surgery^5–7^. Q-switched laser pulses can be produced by using Acousto-optical or Electro-optical modulators (AOM/EOM)^8^ to actively modify the Quality factor within the cavity. However, AOM/EOM consists of bulky and expensive components, as well as the control driver. It will lead to high cost of production and complexity of the Q-switching laser system^9^. In contrast, modulates the cavity Q-factor passively by utilizing the nonlinear optical absorption (NOA) properties of some novel nano-materials may serve as an advanced alternative solution for Q-switched laser pulse generation.

Typically, doped crystals, e.g. Co: MALO^10^ and V: YAG^11^, and semiconductor saturable absorber mirror (SESAM), e.g. InGaAs^12^ are used to induce passively Q-switched laser operation among various commercial laser systems. However, the synthesis process of these commercial saturable absorbers usually involve expensive and complicated doped crystal growth and metal oxide chemical vapor deposition (MOCVD) techniques. Additionally, the narrow operation wavelength band of doped crystals and SESAMs also reduced the corresponding flexibility and applicability^13^. As a result, tremendous research effort has been devoted to searching novel saturable absorber materials which accompany with high operation performance and simple SA fabrication method. The carbon-based nano-materials e.g. graphene^14^, graphene-oxide^15,16^, and carbon nanotube^17^ have attracted great research interests for its demonstrated exceptional nonlinear optical responses. However, these carbon-based materials have their own limitations e.g. lower modulation depth for graphene, diameter control needed for carbon nanotube. The study works on carbon-based materials have inspired the research interests of other layered 2D materials for pulsed laser and nonlinear optical applications.

Transition metal dichalcogenide (TMD), a new class of the 2D family, had gained explosive grow of advertence in the past few years. TMDs provided a wide choice of materials with various favorable properties^18–20^ and which derived extensive applications, for example, photodetectors^21^, biosensors^22^ and energy storage^23^. Layered TMD is composed of stacked planar crystal accompany with a stoichiometry of MX_2_, where M and X stand for the group 4 to 10 transition metal and chalcogen atoms (S, Se, Te), respectively. Remarkably, the modification of NOA properties of TMDs nanosheet can be simply achieved by controlling the corresponding size, thickness^24–26^ and concentration^27^ which provided advance merit for utilizing in nonlinear optical (NLO) devices. In the recent years, the study of Q-switcher application is mainly focusing on the group 6 TMDs materials, such as MoS_2_^28^, MoSe_2_^29^, WS_2_^30^ and WSe_2_^31^, but barely extend to the group 10 TMDs 2D materials. Reported by Zhou *et al*.^32,33^, PtS_2_ had shown a strong interlayer interaction with a bandgap modulated from 0.25 eV to 1.6 eV, which even wider than that of black phosphorus (BP). Meanwhile, the air stability and electron carrier mobility (exceed 1000 cm^2^V^−1^s^−1^)^32^ of group 10 TMDs also had shown superior result. The merits of the Pt-based 2D materials enable some new applications in different fields, e.g. photocatalyst^34^, FETs^33^ and photodetectors^35–38^. Additionally, the Q-switched or mode-locked laser pulses generation have been successfully demonstrated by using PtS_2_^39,40^ and PtSe_2_^41–43^ based saturable absorber. For the infrared pulsed laser and optical communication application, the functional 2D material with a relatively small bandgap is more suitable for the mainstream laser photonic system^41^. Group 6 TMDs (e.g. monolayer WS_2_: 2.1 eV, 590 nm^24^; monolayer MoS_2_: 1.78 eV, 696 nm^41^) have relatively large bandgap and leads to the resonant absorption wavelength located in visible range. Although its bandgap energy can be further modified by changing the layer number, nanosheet size and lattice structure, however, it could further increase the optical loss and reduce stability of the system^41^. Previous studies had shown that monolayer and bilayer PtSe_2_ are indirect bandgap of 1.18 eV and 0.21 eV^44^, respectively (semi-metallic as from 3 layers to bulk form). Remarkably, PtTe_2_ shows the smallest monolayer indirect bandgap of 0.40 eV (~3 µm) among the Pt based TMDs family (it also exhibits semi-metallic as from bilayer to bulk form)^44^. These unique electronic structures of group 10 TMDs allow direct excitation of valence electrons to the conduction band, which it do not require the assistant of intermediate energy state (induced by lattice defect doping) in the band gap^41^. Hence it provided efficient and broadband saturable absorption within the infrared range covering the operational wavelengths of mainstream laser systems. Besides, compared with other 2D material based SAs with near or zero bandgap, such as WTe_2_^45^ or MXene (Ti_3_CN)^46^, PtTe_2_ also shows advancing merits as mentioned previously. WTe_2_ is very susceptible to oxidation in an ambient condition^45,47^, especially during the dispersion process. Usually, an additional protective coating or incorporated within host materials could curb the oxygen corrosion, but it requires additional surface treatment and passivation on the 2D material^48^ which inevitably intervened the nonlinear optical properties of the SA. MXenes materials have the metal atoms that highly exposed on the surfaces^49^. In aqueous solutions of delaminated-MXene, literatures had shown that both the multilayered Ti_3_C_2_T_x_ MXene and monolayer flakes^50^ degrade gradually either in humid air^51^ or water^52^. Meanwhile, to delaminate the MXene, a selective wet etching process is utilized to remove the “A” layer from the MXene MAX phase with followed by intercalation of the guest molecules (e.g. dimethyl sulfoxide -DMSO) and sonication, which highly hazardous Hydrofluoric acid (HF) is used as the wet etchant of the MXene MAX phase^46^. These complicated pre-SA preparation process and degradation problem will taper off the pragmatic application of MXene as a saturable absorber. Combined with the merits of high air stability and electron carrier mobility, group 10 TMDs are able to produce fast and stable nonlinear response to the incident light and generate narrower pulse width for the ultrafast operation^43^, therefore it provided a great potential for various nonlinear optics, pulsed laser and photonic applications.

**Figure 1:**
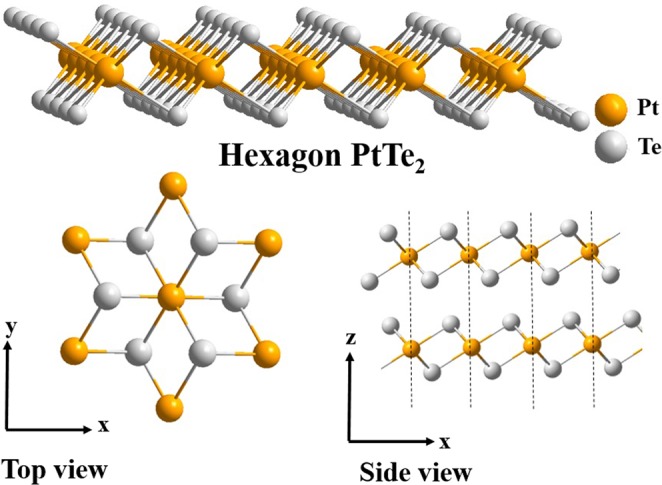
The 3D crystal structure of a monolayer PtTe_2_.

In this work, we have firstly demonstrated the pulsed laser operation by using our homemade Platinum Ditelluride, PtTe_2_ based saturable absorber. The 3D lattice structure of PtTe_2_ is shown in Fig. 1. This newly developed Pt-based TMDs material has strong interlayer interaction^53^ and air stability^54,55^. Contrast to the complicated and expensive CVD fabrication method^53,56,57^, the PtTe_2_ nanosheets used for this experiment are produced by an uncomplicated and cost-effective ultrasonic liquid exfoliation technique and followed by incorporating within the polyvinyl alcohol (PVA) polymer to form a larger-scale PtTe_2_-PVA saturable absorber. The fabrication method employed here is well suitable for making commercial products.

## Result and Discussion

**Figure 2:**
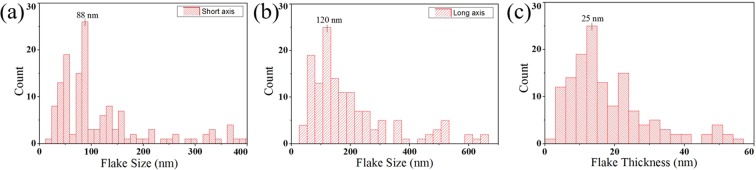
The statistical data of the AFM measurement among 150 PtTe_2_ flakes with respect to the lateral dimensions of (**a**) short axis, (**b**) long axis, and (**c**) thickness of the PtTe_2_ flakes.

**Figure 3:**
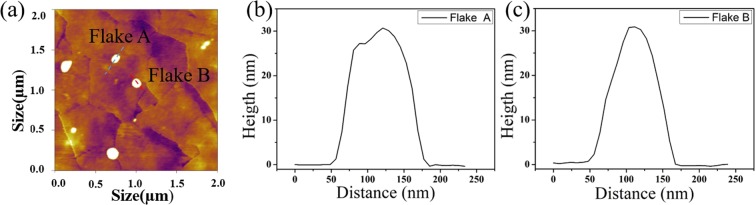
(**a**) AFM image of the fabricated PtTe_2_ flakes. (**b**) The height profile recorded along the blue line of flake A and (**c**) the red line of flake B shown in (**a**).

After successful ultrasonic liquid exfoliation, the morphology, crystal lattice structure and chemical composition of the obtained PtTe_2_ nanosheets were characterized. Figure 2(a–c) show that the most frequently observed dimension along the short-axis, long-axis and thickness are around 88 nm, 120 nm, and 25 nm, respectively. Topology graph of two randomly selected flakes and their corresponding height profiles along the marked lines are shown in Fig. 3. The lateral dimensions for long-axis of both flake A and flake B are around 125 nm, which within the statistic result and indicated the successful exfoliation of the PtTe_2_ sample.

**Figure 4:**
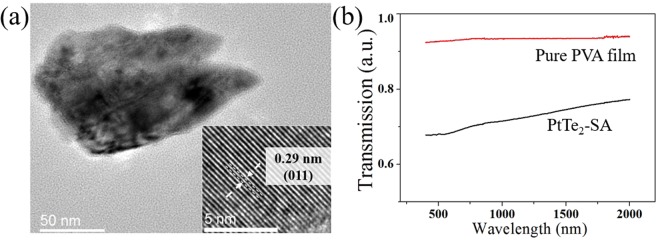
(**a**)The FETEM image of a PtTe_2_ flake with inserted corresponding high-resolution FETEM image and (**b**) the Transimmsion spectrum of the PtTe_2_-SA.

**Figure 5:**
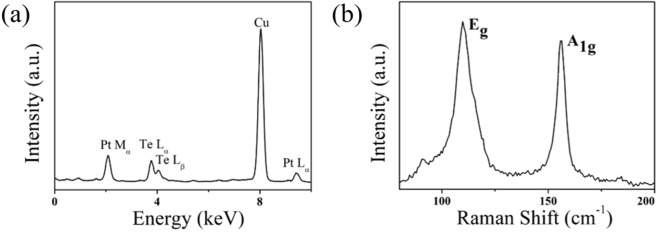
(**a**) The EDS spectrum and (**b**) Raman spectrum of the prepared PtTe_2_ sample.

From the TEM image (Fig. 4(a)), it shows that the lateral size of a randomly selected PtTe_2_ flake well match with the AFM statistical results. The relevant high-resolution FETEM image is inserted in Fig. 4(a) and shows an inter-plane distance of 0.29 nm corresponding to the (011) plane of PtTe_2_^58^. The Transmission spectra of the PtTe_2_-PVA composite and a reference PVA film are presented in Fig. 4(b) which shows that the type-II Dirac semimetal^57^ PtTe_2_ has no characteristic absorption peak from 500 nm to 2000 nm wavelength range, as similar to graphene-SA^59^. For the chemical composition of PtTe_2_ sample, Fig. 5(a) shows only characteristic peaks from Pt or Te and no other observable impurities is found (Cu peak is induced from the copper mesh support for TEM measurement). Meanwhile, as shown in Fig. 5(b), the Raman-active modes E_g_ and A_1g_ were positioned at 110.7 cm^−1^ and 154.8 cm^−1^, respectively, and which agree well with the previous report^57^. The E_g_ mode is representing the in-plane vibration mode of Platinum and Ditelluride atoms. Meanwhile, the A_1g_ is standing for the out-plane vibration modes of Ditelluride atoms. These strong characteristic Raman peaks reveal that the fabricated PtTe_2_ nanosheets possess with good crystallinity.

**Figure 6:**
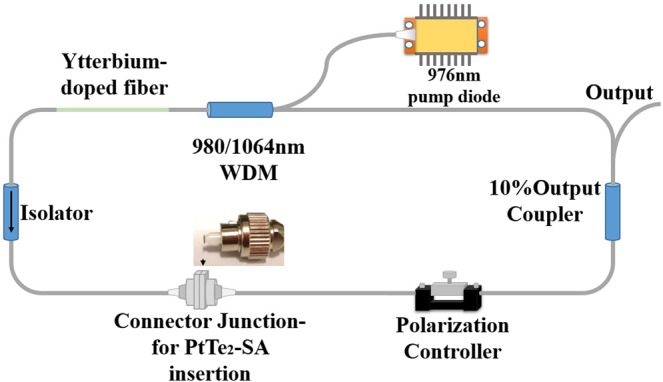
The schematic experimental setting of the passive Q-switching Yb-doped fiber laser.

For the laser test, a pure Polyvinyl alcohol (PVA) film, which possess of identical thickness as the PtTe_2_-PVA SA film, was first inserted between the connector junction of the ring cavity as shown in Fig. 6 to act as a control experiment set. No Q-switched laser pulses were observed by modifying the polarization direction of the polarization controller (PC) and pumping power. It confirms Q-switching operation cannot be obtained by using pure PVA polymer or cavity phase self-modulation. Further on, the fabricated PtTe_2_-SA was cut with a size in approximately 1 mm × 1 mm, and was introduced into the laser system by sandwiching between two fiber connectors. The parasitic reflection could be eliminated by using FC/APC connector and thus improved the stability of the Q-switching system.

**Figure 7:**
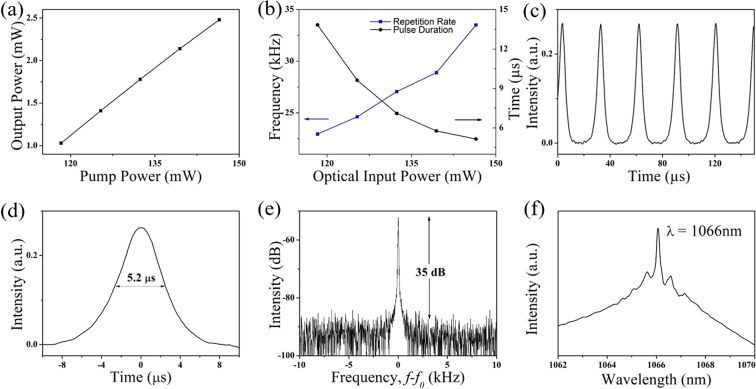
Q-switched laser performance: (**a**) The average output power and (**b**) Repetition rate and Pulse duration variation with respect to the different pump power. (**c**) Pulse train, (**d**) Single pulse profile, (**e**) Radio frequency spectrum, and (**f**) Wavelength spectrum corresponding to the maximum output single pulse energy of 74.0 nJ.

Stable Q-switched pulse train was observed as the pump power elevated to the starting threshold of 118 mW. However, when the pump power raised beyond 146.5 mW, the Q-switched output was vanished due to the over saturation of the absorber at high input fluence^26,60^. The average output power was measured as from 1.03 mW to 2.48 mW with respect to the pump power from 118 mW to 146.5 mW as shown in Fig. 7(a). The modulation ranges of the pulse width (Full width at half maximum) and the repetition rate are 13.6 μs to 5.2 μs and 23.0 kHz to 33.5 kHz, respectively as shown in Fig. 7(b). The fluctuation of repetition rate and output power of the Q-switched laser are less than 5% within the test time of about 30 to 45 minutes during the measurement. The minimum pulse duration can be further shortened by either enhancing the modulation depth of the SA or reducing the total length of ring cavity^61^. The maximum recorded single pulse energy is about 74.0 nJ which is comparable to previous literature of passive Q-switching Yb-doped fiber laser with utilizing another TMDs based SA, for instance, MoSe_2_ (116 nJ)^29^, MoS_2_ (126 nJ)^28^, WS_2_ (13.6 nJ)^30^, and even the group 10 PtS_2_ (45.6 nJ) Q-switched laser operated in 1569 nm^39^. Meanwhile, the correlated pulse train, single laser pulse profile, radio-frequency (RF) (resolution bandwidth: 20 Hz) and wavelength spectra has shown in Fig. 7(c–f) corresponding to the maximum output single pulse energy of 74.0 nJ.

The passively Q-switched Yb-doped fiber laser based on PtTe_2_ saturable absorber was first demonstrated. A stable Q-switched operation was illustrated from 1066 nm fiber ring cavity. The achieved pulse duration and repetition rate range are 13.6 μs to 5.2 μs and 23.0 kHz to 33.5 kHz, respectively, with correlated to the average output power from 1.03 mW to 2.48 mW. The highest achieved single pulse energy is 74.0 nJ which is well comparable with the output achieved by using other group 6 or 10 TMDs saturable absorber within the fiber laser system. This first demonstration proves the novel photonic applications of the newly developed 2D PtTe_2_ material.

## Methods

### PtTe_2_ nanosheets preparation

The PtTe_2_ nanosheets were prepared by using ultrasonic liquid exfoliation to break the interlayer bonding. Isopropyl Alcohol (IPA) was selected as the exfoliation solvent in order to prevent flakes aggregation because of its well surface energy compatibility with the TMDs substances. First, 175 mg PtTe_2_ powder, which purchased from Six Carbon Inc., was mixed with 175 ml IPA solvent. Then, the mixture was probe sonicated for (SCIENTZ-1200E, Ningbo Scientz Biotechnology Co., Ltd) over 15 hours. The probe sonication operated at the frequency of 20 kHz and power of 1500 W. Then the PtTe_2_ suspension was centrifuged at 3000 rpm speed for 5 minutes and only the supernatant was extracted to remove large flakes and impurities.

### Sample characterizations

The statistic measurement of the size and thickness distribution among 150 flakes were obtained by utilizing an atomic force microscope (Bruker Nanoscope 8). The Ultrahigh magnification image and chemical ingredient of the PtTe_2_ flakes were obtained by using field emission transmission electron microscopy (FETEM. JEM-2100F) with an accessory of energy-dispersive X-ray spectroscopy (EDS). Raman spectra was measured by a LabRAM HR 8000 Raman Spectrometer.

### PtTe_2_ saturable absorber preparation

In order to synthesize PtTe_2_-SA, firstly, PVA solution with 15% weight percentage was prepared by mixing the pure PVA powders with deionized water. Then the fabricated PtTe_2_ suspension was added into the PVA solution by a weight ratio of 1: 6 and further stirred for 30 minutes. Afterward, the mixture was dried under 60 °C for 48 hours in the oven to form a PtTe_2_-PVA composite thin film and it serves as a transmission type saturable absorber for the Q-switching experiment.

### Experimental setup

A ring cavity of Ytterbium-doped fiber laser was built as the schematic diagram shown in Fig. 6 for testing the performance of PtTe_2_-SA. The cavity was constituted with a 980/1064 wavelength division multiplexer (WDM), a 0.68 m long Yb-doped single mode fiber (LIEKKI Yb1200-4/125), a polarization-independent isolator (PI-ISO), a polarization controller (PC), and an output coupler with 90:10 coupling ratio. The total cavity length is about 12 meters. The fabricated PtTe2-SA was cut with a size in approximately 1 mm × 1 mm, and was introduced into the cavity by sandwiching between two fiber connectors.
